# Health-Related Quality-of-Life Measures in Patients with Heart Failure Cardiogenic Shock Following Axillary Mechanical Circulatory Support

**DOI:** 10.3390/medsci13030146

**Published:** 2025-08-19

**Authors:** Hans Mautong, Aarti Desai, Shriya Sharma, Jose Ruiz, Juan Leoni, Rohan Goswami

**Affiliations:** 1 School of Health, Universidad Espíritu Santo-Ecuador, Samborondón 092301, Guayas, Ecuador; hansmautong@uees.edu.ec; 2 John H. Stroger Jr. Hospital of Cook County, Chicago, IL 60612, USA; 3 Division of Heart Failure and Transplantation, Mayo Clinic, 4500 San Pablo Road, Jacksonville, FL 32224, USA; desai.aarti@mayo.edu (A.D.); ruizmorales.jose@mayo.edu (J.R.); leoni.juan@mayo.edu (J.L.); 4 St. Elizabeth Medical Center, Boston, MA 02135, USA; shriyasharmap@gmail.com

**Keywords:** quality of life, the Impella, heart failure, cardiogenic shock, KCCQ, KCCQ-12, mechanical circulatory support

## Abstract

**Background:** Patients with end-stage heart failure-related cardiogenic shock (HF-CS) are conclusively associated with a poor health-related quality of life (HRQL). Axillary mechanical circulatory support (aMCS), such as the Impella 5.5, is increasingly used in this population and may improve HRQL during hospitalization by providing enhanced left ventricular unloading. We aimed to assess changes in HRQL between admission and two weeks after Impella 5.5 placement in patients with HF-CS, using the Kansas City Cardiomyopathy Questionnaire (KCCQ). **Methods:** We conducted a prospective longitudinal analysis on patients with the Impella 5.5 between May 2023 and July 2023. Participants completed the condensed KCCQ-12 at admission and again two weeks post-implantation. Changes in the scores were evaluated using the Wilcoxon signed-rank test. **Results:** Fifteen patients were enrolled. The median age was 59 years (50–63), and the median ejection fraction at implantation was 20% (15–30). On admission, most patients reported an overall HRQL of poor-to-fair (46.7%) according to the summary KCCQ-12 score. The median overall summary score increased significantly after Impella 5.5 support (50.52 vs. 28.13, *p* = 0.005). Symptom frequency (70.83 vs. 43.75, *p* = 0.009) and quality-of-life (50.00 vs. 12.50, *p* = 0.023) domains improved significantly, while physical limitation showed a positive trend and social limitation remained unchanged. These HRQL improvements occurred alongside a significant shift toward lower SCAI shock stages, marked increases in cardiac output and cardiac index, and no escalation in vasoactive-inotropic requirements. **Conclusions:** Impella 5.5 support in HF-CS patients was associated with early and clinically meaningful improvements in HRQL, particularly in symptom frequency and quality of life, during the critical pre-transplant or recovery period. These findings suggest that the Impella 5.5 may provide both physiological and patient-perceived benefits in this high-risk population.

## 1. Background

Heart failure (HF) patients experience significantly diminished health-related quality of life (HRQL) compared to individuals with other chronic diseases or those who are healthy. HRQL represents how patients subjectively perceive the impact of their clinical condition on their overall well-being and can vary widely among individuals with the same disease [1]. HF patients face various physical and emotional symptoms, including dyspnea, fatigue, edema, sleep difficulties, depression, and chest pain [1]. These symptoms limit their daily physical and social activities, resulting in a decline in HRQL [1]. Interestingly, poor HRQL has been closely associated with higher rates of hospitalization and mortality [2]. Thus, it is essential to recognize that HRQL is a subjective measure that extends beyond objective clinical or physiological measures and significantly impacts the lives of HF patients.

While significant progress has been made in developing treatments for chronic HF, the management of acute decompensated heart failure (ADHF) remains a challenge, especially considering its profound impact on HRQL. The sickest patients on the HF spectrum are those with heart failure cardiogenic shock (HF-CS), who often require advanced treatment options such as axillary mechanical circulatory support (aMCS) and experience changes in their quality of life [3]. Among the devices utilized for temporary aMCS, the Impella 5.5 (Abiomed, Danvers, CA, USA) device has shown promise in offering enhanced support for these patients and has been shown to improve clinical outcomes [4]. This intravascular microaxial blood pump is designed to temporarily assist the left ventricle with enhanced flow rates of up to 5.5 L/min, thereby reducing the workload of the left ventricle, improving systemic circulation and end-organ perfusion, and leading to functional improvements [5,6]. Despite its potential benefits, data regarding the impact of aMCS on HRQL have not been reported in the HF-CS population.

Patient-reported outcomes measures (PROMs) offer a systematic way to collect patients’ perspectives on their health status. One such standardized tool used to assess the HRQL measures of HF patients is the Kansas City Cardiomyopathy Questionnaire (KCCQ-23) [7]. The KCCQ-23 was developed collaboratively, with input from both patients and clinicians, to assess the impact of heart failure on patients’ quality of life [8]. To facilitate its implementation in routine clinical care, the KCCQ-23 was condensed from its original 23 items to a 12-item version (KCCQ-12) [9]. Recent work has explored strategies to optimize outcomes in advanced HF patients receiving mechanical circulatory support, which may indirectly influence quality of life. For example, structured telemedicine, remote monitoring, and continuous hemodynamic assessment during device support have been shown to maintain clinical stability and reduce hospitalizations [10,11]. Such approaches highlight the importance of close monitoring and timely intervention during mechanical support, supporting our focus on HRQL in HF-CS following aMCS with the Impella 5.5.

Our study aims to evaluate the changes in HRQL measures among patients with HF-CS following Impella 5.5 placement by analyzing the difference between the KCCQ-12 scores recorded at admission and two weeks post-device placement. To our knowledge, this is the first study to specifically assess the changes in HRQL outcomes among HF-CS patients supported by the Impella 5.5.

## 2. Methods

### 2.1. Study Design, Population, and Data Collection

After IRB approval, we conducted a prospective longitudinal study at the Mayo Clinic, Florida, enrolling patients with severe ADHF admitted between May 2023 and July 2023. The study included patients requiring axillary mechanical circulatory support (aMCS) using the Impella 5.5 device as a bridge to transplant (BTT). All participants were classified as New York Heart Association (NYHA) functional class IIIb or IV and were receiving single or dual inotropic therapy before device implantation for hemodynamic optimization. Patients who received other forms of mechanical circulatory support were excluded.

The KCCQ-12 was administered to patients with HF-CS on admission and 2 weeks after the placement of the Impella 5.5. Patients’ responses to the questionnaire were tabulated using REDCap (Research Electronic Data Capture), a secure, web-based platform designed to support data capture for research studies. All participants had the device at the time when the responses for the follow-up questionnaire were collected. Baseline characteristics were extracted from the electronic medical record.

### 2.2. KCCQ-12 Administration and Scoring

**Figure 1:**
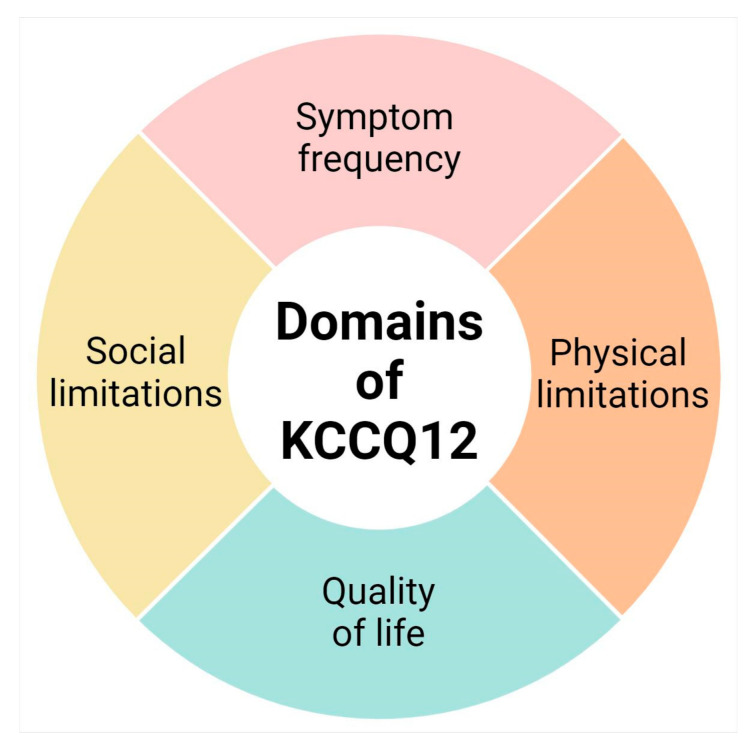
Domains of the condensed KCCQ-12 [13].

The KCCQ-12 is a validated 12-item self-reporting questionnaire that was specifically designed to assess HRQL in patients with HF [9].The questionnaire has a recall period of two weeks to account for the day-to-day variability in heart failure symptoms [9]. Although this PROM is a condensed version of the original KCCQ-23, the KCCQ-12 correlates highly with the original scales (>0.93) in all clinical settings, shows high test-retest reliability (>0.76), and is responsive to clinical changes [12]. Each of the 12 items is answered on a Likert scale, and the responses are then grouped and used to calculate a score for four different domains: physical limitation, symptom frequency, quality of life, and social limitation (Figure 1) [9].

Each domain has its own individual score on a scale of 0–100; higher scores indicate a better health status. Additionally, the overall summary score was calculated as the average of the score of all four domains [9]. Depending on the domain, if a sufficient number of items were missing, the score for that domain was not calculated. Based on the score, individuals were classified between 25-point ranges [9]. Scores from 0 to 24: very-poor-to-poor; 25 to 49: poor-to-fair; 50 to 74: fair-to-good; and 75 to 100: good-to-excellent [9].

### 2.3. Statistical Analysis

All statistical analyses were performed using SPSS for Windows (version 23.0; SPSS Inc., Chicago, IL, USA). The Kolmogorov–Smirnov test was used to test for the normality of distribution. Categorical variables were presented as percentages. Continuous variables were presented as medians with interquartile ranges. The chi-square test was used to compare the frequencies of pre-Impella and post-Impella use of inotropes and vasopressors, as well as the rate of SCAI stage. The Wilcoxon signed-rank test was used to compare the hemodynamic parameters and the KCCQ-12 scores at baseline and 2 weeks post-Impella 5.5 placement. The Wilcoxon signed-rank test was selected due to the ordinal nature and non-normal distribution of the KCCQ-12 scores. This non-parametric test is suitable for comparing paired, non-normally distributed data and was appropriate for our study’s pre- and post-intervention design. Statistical significance was defined by a two-tailed *p* < 0.05.

### 2.4. Ethical Considerations

This study was conducted in accordance with the Declaration of Helsinki and was approved by the Mayo Clinic Institutional Review Board. Informed consent was obtained for all participants, and personal data protection was ensured by maintaining patient confidentiality and anonymity.

## 3. Results

During the study period, 15 HF-CS patients underwent placement of the Impella 5.5 device as a BTT. All of them survived the procedure and were able to answer the follow-up questionnaire 2 weeks after the device placement. All fifteen patients were enrolled in our study.

### 3.1. Baseline Clinical and Demographic Characteristics

Most of the patients were males (80%), and their median age was 59 (50–63) years. The proportion of African American and white races was equally distributed (46.7%). About 46.7% of patients had hypertension and up to 60% had diabetes. Former smokers represented about 46.7% of our study population. The median glomerular filtration rate was 62 (45–71), and about 13.3% of patients had CKD stage 4. Regarding the etiology of their heart failure, most patients had non-ischemic heart failure (80%) and were classified as NYHA class IV (47%). The median ejection fraction (EF) before Impella 5.5 placement was 20% (15–30).

The median device-to-transplant time was 35 (15–51) days. Similarly, the median ICU length of stay after transplant was 6 (5–7) days, while the median total hospital length of stay was 60 (52–84) days (Table 1).

### 3.2. Inotrope and Vasopressor Requirements and Shock Severity Pre- and Post-Impella 5.5 Implantation

Pre-Impella inotrope/vasopressor use is summarized in Table 2. Following Impella 5.5 implantation, there were no statistically significant changes in the proportion of patients receiving dobutamine (73.3% pre- vs. 60.0% post-implantation; *p* = 0.439), milrinone (60.0% vs. 66.7%; *p* = 0.705), vasopressin (6.7% vs. 6.7%; *p* = 0.999), epinephrine (13.3% vs. 6.7%; *p* = 0.543), or norepinephrine (6.7% vs. 0%; *p* = 0.309). Similarly, the vasoactive-inotropic score (VIS) remained unchanged post-Impella 5.5 implantation, 5 (3.75–10) vs. 5 (3.75–10), *p* = 0.307.

In contrast, a significant improvement in shock severity was observed, with the majority of patients improving to SCAI stage B post-implantation (86.7% vs. 13.3% pre-implantation), and a marked reduction in the proportion of patients in SCAI stage D (6.7% vs. 60.0% pre-implantation; *p* = 0.001).

### 3.3. Hemodynamic Changes Pre- and Post-Impella 5.5 Implantation

Using the Wilcoxon signed-rank test, no statistically significant changes were observed in heart rate, blood pressure parameters (systolic, diastolic, mean arterial pressure, and pulse pressure), right atrial pressure, pulmonary artery pressures (systolic, diastolic, and mean), or pulmonary capillary wedge pressure 72 h after Impella 5.5 implantation compared to baseline (*p* > 0.05 for all) (Table 3).

In contrast, both Fick cardiac output and cardiac index demonstrated significant improvements post-implantation, increasing from a median of 3.67 L/min (3.10–3.90) to 5.70 L/min (4.80–7.20), *p* = 0.001, and from 1.70 L/min/m^2^ (1.50–1.95) to 2.80 L/min/m^2^ (2.50–3.00), *p* = 0.001, respectively.

### 3.4. KCCQ-12 Scores on Admission and 2 Weeks After Impella 5.5 Placement

The answers to each item of the KCCQ-12 in the original order at baseline and 2 weeks after Impella 5.5 placement are presented on Supplementary Table S1. After the placement of the Impella 5.5, about 60% of patients experienced an improvement in their overall summary score, 40% of which were very large improvements (>20 points), as shown in Supplementary Table S2. Similarly, regarding the physical limitation domain, 61.5% of patients experienced some degree of improvement, the majority of which were very large improvements (23.1%). When analyzing the symptom frequency domain, most patients (66.7%) experienced an improvement in their scores, which in most cases was very large (53.3%). In the quality-of-life domain, about 73.4% of patients experienced an improvement in their scores, which was very large in most of them (46.7%). Finally, when assessing the social limitation domain, most patients experienced a deterioration or no improvement (53.9%).

**Figure 2:**
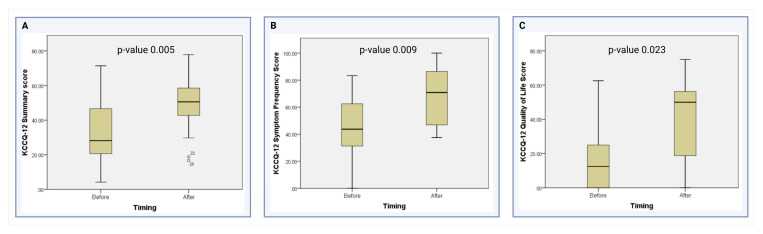
Comparison of the KCCQ-12 scores in the significant domains [13]. (**A**) Comparison of KCCQ-12 Summary scores before and after Impella 5.5 placement. (**B**) Comparison of KCCQ-12 Symptom Frequency scores before and after Impella 5.5 placement (**C**) Comparison of KCCQ-12 Quality of Life domain scores before and after Impella 5.5 placement.

As shown in Table 4, the median KCCQ-12 overall summary score after the placement of the Impella 5.5 was significantly higher than before the device (50.52 vs. 28.13, *p* = 0.005) (Figure 2A). When analyzing the KCCQ-12 domains, the median scores in the symptom frequency domain (70.83 vs. 43.75, *p* = 0.009) (Figure 2B) and the quality-of-life domain (50 vs. 12.5, *p* = 0.023) significantly improved after device placement (Figure 2C). The physical limitation domain showed a non-significant trend towards improvement after device placement (62.50 vs. 50, *p* = 0.293). Finally, there was no difference in the social limitation domain when comparing patients before and after the placement of the Impella 5.5 (25 vs. 25, *p* = 0.342) at our institution.

Supplementary Table S3 provides further details on the changes in percentages of KCCQ-12 classifications at baseline and 2 weeks post-Impella 5.5 placement. On admission, most patients reported they were overall feeling poor-to-fair (46.7%) according to their total KCCQ-12 score. After the Impella 5.5 placement, most of them reported feeling fair-to-good (40%). The same trend was observed among all the domains of the questionnaire, as the percentage of people feeling fair-to-excellent began to rise and the percentage of people feeling very-poor-to-fair began to decline after being mechanically supported. More specifically, after the Impella 5.5 placement, the majority of people improved from feeling poor-to-fair (40%) to good-to-excellent (46.7%) in the symptom frequency domain, and from very-poor-to-poor (60%) to fair-to-good (46.7%) in the quality-of-life domain.

## 4. Discussion

**Figure 3:**
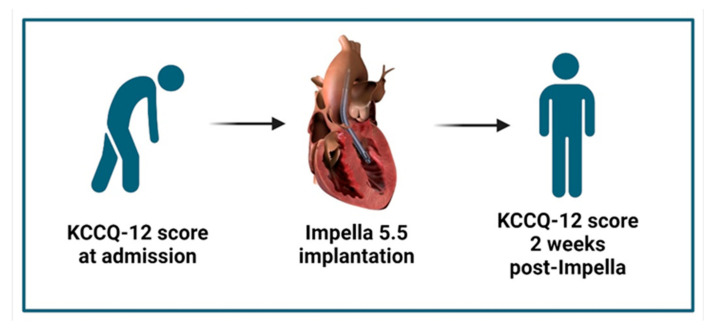
Improvement in pre-and post-Impella 5.5 placement KCCQ score [13].

Our analysis of the KCCQ-12 scores at baseline and 2 weeks post-Impella 5.5 placement demonstrates a significant improvement in the overall HRQL of patients with HF. In particular, the scores of the symptom frequency and quality-of-life domains significantly increased, despite hospitalization and management for cardiogenic shock. Our analysis also demonstrates a non-significant but positively trending improvement in physical limitations. These findings reveal the potential of the Impella 5.5 in enhancing the well-being of critically ill HF-CS patients with profoundly reduced HRQL measures at baseline. Beyond providing hemodynamic support, the device may help mitigate the physical and emotional burdens often associated with organ replacement therapy (Figure 3).

### 4.1. PROMs in Assessing QOL While Hospitalized

There is no standardized method of assessing HRQL measures in the HF-CS population currently. The New York Heart Association functional class is commonly used to assess health status; however, the NYHA HF classification assesses only the HF symptoms pertaining to physical activity [14]. This often differs from how patients perceive the severity of their overall quality of life.

PROMs offer a systematic way to collect patients’ perspectives on their health status, providing more detailed information and better predicting prognosis [7]. The KCCQ-12 is a PROM that evaluates symptom frequency, burden, physical and social limitations, and quality of life in HF patients [9]. It is widely utilized and extensively validated in the HF population, providing not only a summary of health status but also assessing responsiveness to HF treatment and serving as a reliable predictor of clinical risk [15].

The KCCQ-12 has proven to be a valid, reliable, responsive, and prognostically important measure of patients’ health status for those with HFrEF. The association of KCCQ-12 scores with prognosis has been demonstrated at the time of discharge from hospitalization for ADHF [16]. Furthermore, it has previously been used to assess the quality of life of patients with advanced HF who have undergone left ventricular assist device (LVAD) placement [17,18].

### 4.2. The Role of Impella 5.5 in the Improvement in KCCQ-12 Scores in the Four Domains

In addition to the HRQL findings, our analysis of inotrope and vasopressor utilization, SCAI shock stage, and invasive hemodynamic parameters pre- and post-Impella 5.5 implantation provides important physiologic context. We observed a significant shift toward less severe shock, with a majority of patients improving to SCAI stage B within 72 h of implantation. This was accompanied by a marked increase in cardiac output and cardiac index, as well as reductions in right- and left-sided filling pressures, although not all changes reached statistical significance. Importantly, these improvements in shock severity occurred without a significant change in the vasoactive-inotropic score, suggesting that the observed hemodynamic and clinical gains were achieved in the absence of escalating pharmacologic support. The hemodynamic improvements therefore likely contributed, independently, to the observed symptomatic relief and enhanced quality-of-life scores, underscoring the dual benefit of early Impella 5.5 support—leading to physiological and patient-reported benefits.

Between baseline and 2 weeks post-Impella, over 60% of patients experienced an improvement in their overall summary scores, with a significant proportion showing an improvement of over 20 points. These findings are in line with the broader understanding highlighted by Johansson et al., who conducted a large-scale study involving 23,291 patients from eight different world regions. They found a significant association between lower KCCQ-12 scores and increased risk of adverse outcomes, with an adjusted hazard ratio of 1.18 for death per each 10-unit decrement in KCCQ-12 summary score (95% CI, 1.17–1.20) [19]. Furthermore, their research revealed that lower HRQL was a strong and independent predictor of all-cause death and heart failure hospitalization across various geographic regions, symptom severities, and ejection fraction categories [19]. This highlights the importance of incorporating such assessments into clinical practice for improved patient management and risk stratification, especially in patients with ADHF or HF-CS, as these patients have a poor HRQL and increased mortality.

Our results highlight the capability of a temporary aMCS device like the Impella 5.5 to improve the HRQL of patients with HF-CS. In our study, this improvement in the overall HRQL was mainly seen in the symptom frequency and quality-of-life domains. These findings suggest that the Impella 5.5 not only supports hemodynamic function but also plays a key role in alleviating the debilitating symptoms and emotional burdens often experienced by critically ill HF-CS patients.

***Symptom frequency:*** Our results reveal a significant improvement in the symptom frequency domain of the KCCQ-12 after 2 weeks of Impella 5.5 placement (Figure 3). By reducing the workload of the heart and improving systemic circulation, the Impella 5.5 offers a promising therapeutic strategy for HF patients, particularly those in critical conditions such as HF-CS. This finding suggests that the mechanical circulatory support provided by the Impella device effectively augments cardiac output, thereby alleviating symptoms such as dyspnea, fatigue, and exercise intolerance [20]. These symptomatic improvements are consistent with previous research highlighting the beneficial effects of temporary aMCS in relieving hemodynamic compromise and enhancing patient comfort, which Shapiro et al. and Zaky et al. have previously described [21,22].

***Quality of Life:*** Our results demonstrated a significant improvement in the quality-of-life domain of the KCCQ-12 following two weeks of Impella 5.5 placement. This positive change may reflect the device’s ability to alleviate the severe physical and emotional burdens associated with HF-CS [20]. By enhancing cardiac output and relieving symptoms such as fatigue and breathlessness, patients experienced a noticeable recovery in their overall sense of well-being. Improved perfusion may also mitigate the cognitive effects often linked to low cardiac output, such as “brain fog,” further facilitating meaningful gains in patients’ perceived quality of life [20]. These findings emphasize the value of the Impella 5.5 not only as a life-sustaining therapy but also as a means to restore a sense of normalcy and improve day-to-day living for these patients.

***Physical Limitations:*** Our data reveal a positively trending improvement in physical capabilities following Impella 5.5 implantation. This may be attributed, in part, to our institutional protocol for early ambulation, typically within 24 h. Our program works with both physical and occupational therapy to enhance the utility of mobility in whole-body optimization prior to organ replacement therapies. Patients ambulate daily, track their progress, and compete amongst each other to complete ‘the most laps’ in the ICU while on Impella support. Our experience has demonstrated shorter post-transplant length-of-stay in both the ICU and the hospital in patients who underwent Impella 5.5 placement as BTT [21]. The small sample size may account for the non-significant *p*-value observed.

***Social Limitations:*** Interestingly, although significant improvements were observed in several KCCQ-12 domains post-Impella 5.5 placement, the social limitation domain did not show substantial change. This discrepancy is likely due to the patients’ hospitalized status during the duration of our study, which inherently limits the social interactions and activities of patients with HF [23]. As these patients remained in a monitored, clinical setting, they likely had limited opportunities for social engagement outside of the hospital, impacting their perceived social limitations. The absence of improvement in the social limitation domain likely reflects the inpatient setting, which inherently restricts social engagement despite physiological recovery. Future assessments after discharge are needed to determine whether social functioning improves as patients resume living in their normal environments. In the interim, structured in-hospital engagement initiatives may help mitigate perceived social limitations during prolonged aMCS. Since the gathering of this data, however, we have had several nursing-led initiatives to improve patient social satisfaction—including game nights where patients gather in common areas and interact with each other, playing cards or celebrating special events or holidays.

### 4.3. Implications for Clinical Practice

Orthotopic heart transplantation stands as the primary treatment for end-stage HF, often referred to as the “gold standard.” For patients with advanced heart failure who are not responding to medical therapy, heart transplantation remains the sole effective therapy, boasting excellent short- and long-term survival rates estimated at 85% at one year and 50% at ten years [24]. The observed enhancements in HRQL and symptom management have important implications for the clinical management of HF-CS patients. The ability of Impella 5.5 placement to rapidly ameliorate symptoms and improve functional status emphasizes its role as a valuable therapeutic intervention in the acute care setting.

These observations are consistent with recent evidence from Matteucci et al. and Mariani et al., which—although not directly measuring PROMs—demonstrate that structured monitoring and timely intervention can have important implications for patient well-being during mechanical circulatory support [10,11]. Matteucci et al. showed that, during the COVID-19 pandemic, telemedicine and remote monitoring preserved clinical stability and reduced avoidable hospitalizations in cardiac device patients, an approach that may help sustain functional capacity and minimize symptom exacerbations that undermine quality of life [10]. Likewise, Mariani et al. demonstrated that continuous pulmonary artery pressure monitoring in LVAD patients was feasible and enabled early hemodynamic optimization, potentially preventing decompensation and supporting day-to-day health status [11]. Applying these principles to HF-CS patients supported by the Impella 5.5 could help preserve HRQL by maintaining stability, preventing clinical deterioration, and enabling recovery during the critical pre-transplant period, aligning with our observed improvements in KCCQ-12 scores within two weeks of device implantation.

Clinicians should consider the early implementation of temporary aMCS devices like the Impella 5.5 in HF-CS patients to optimize outcomes and mitigate the risk of adverse events associated with hemodynamic instability. Overall, these results emphasize the significance of aMCS with the Impella 5.5 in managing advanced HF patients awaiting heart transplantation. Based on published data via the Scientific Registry of Transplant Recipients (SRTR), after implementing our Impella 5.5 program for early escalation in cardiogenic shock patients failing standard inotrope and medical therapy, our survival in the Impella-specific cohort has remained excellent, with all 15 of these patients surviving to, and 1 year post-, transplantation [25].

## 5. Limitations

The relatively small sample size of our study, comprising only 15 patients, may limit the generalizability of the findings and the ability to detect statistically significant differences in all KCCQ-12 domains. In addition, the observational design of the study prevents us from establishing a causal relationship from the observed associations. Therefore, further research involving larger multicenter cohorts is needed to validate these findings and improve our understanding of how the Impella 5.5 device can improve HRQL and clinical outcomes in patients with severe ADHF and HF-CS. In addition, only patients clinically able to complete the 2-week KCCQ-12 were included, introducing potential selection bias that could overestimate HRQL improvements. Furthermore, our study did not include a control group or sham procedure arm. Follow-up in our cohort was limited to 14 days for multiple reasons: patients undergoing transplants, the FDA’s indication on timeframe, and need for support staff for collecting surveys in a timely fashion. This finding highlights the need for longer-term assessment in further studies.

## 6. Conclusions

The impact of aMCS with Impella 5.5 extends beyond hemodynamic support for patients with HF-CS. Our findings highlight its potential to significantly improve HRQL in this critically ill population. Within just two weeks of receiving Impella 5.5 support, patients demonstrated marked improvements in overall health status, symptom frequency, and quality of life as measured by the KCCQ-12. This intervention has the potential to restore vitality and enhance cardiac function, promising a better HRQL for this patient population.

## Figures and Tables

**Table 1:** Baseline clinical and demographic characteristics of the study population.

Parameter	n = 15
**Males** (n, %)	12 (80)
**Age** (median, IQR)	59 (50–63)
**Race** (n, %)	
- African American	7 (46.7)
- White	7 (46.7)
- Other	1 (6.7)
**Comorbidities**	
- Hypertension (n, %)	7 (46.7)
- Diabetes Mellitus (n, %)	9 (60)
- Smoker (n, %)	
- Never smoked	8 (53.3)
- Former smoker	7 (46.7)
- Chronic Kidney Disease (CKD) stages (n, %)	
- CKD Stage 4	2 (13.3)
- CKD Stage 3b	1 (6.7)
- CKD Stage 3a	4 (26.7)
- CKD Stage 2	6 (40)
- Glomerular Filtration Rate (GFR) (mL/min/m^2^) (median, IQR)	62 (45–71)
- Body Mass Index (BMI) (kg/m^2^)	
- BMI (median, IQR)	27.74 (24.90–28.60)
- BMI over >30 kg/m^2^ (n, %)	3 (20)
**Etiology** (n, %)	
- Ischemic cardiomyopathy	3 (20)
- non-Ischemic cardiomyopathy	12 (80)
**Baseline ejection fraction (EF) (%)** (median, IQR)	20 (15–30)
**Baseline NYHA staging** (n, %)	
- NYHA 3B	8 (53)
- NYHA 4	7 (47)
**Device-to-transplant duration** (days) (median, IQR)	35 (15–51)
**ICU length-of-stay** (days) (median, IQR)	6 (5–7)
**Total hospital length-of-stay** (days) (median, IQR)	60 (52–84)

Abbreviations: BMI, body mass index; CKD, chronic kidney disease; EF, ejection fraction; GFR, glomerular filtration rate; NYHA, New York Heart Association. Data are median (IQR) or n (%).

**Table 2:** Inotrope and vasopressor utilization and SCAI shock stage pre- and post-Impella 5.5 implantation.

	Pre-Impella (n, %)	Post-Impella (n, %)	*p*-Value
Dobutamine	11 (73.3%)	9 (60.0%)	0.439
Milrinone	9 (60.0%)	10 (66.7%)	0.705
Vasopressin	1 (6.7%)	1 (6.7%)	0.999
Epinephrine	2 (13.3%)	1 (6.7%)	0.543
Norepinephrine	1 (6.7%)	0 (0.0%)	0.309
SCAI Stage			
- SCAI B	2 (13.3%)	13 (86.7%)	0.001
- SCAI C	4 (26.7%)	1 (6.7%)	
- SCAI D	9 (60.0%)	1 (6.7%)	

Abbreviations: SCAI, Society for Cardiovascular Angiography and Interventions shock stage; Data are n (%). *p*-values by chi-square test; N = 15.

**Table 3:** Comparison of hemodynamic parameters pre- and post-Impella 5.5 Implantation.

	Pre-Impella	Post-Impella (72 h)	*p* Value
Heart Rate (bpm)	93 (84–112)	92 (84–110)	0.777
Systolic BP (mmHg)	104 (98–120)	99 (96–119)	0.232
Diastolic BP (mmHg)	75 (73–82)	76 (70–81)	0.776
Pulse pressure (mmHg)	26 (26–34)	23 (18–40)	0.255
Mean Arterial Pressure (MAP) (mmHg)	86 (81–97)	85 (79–93)	0.395
Right Atrial (RA) pressure (mmHg)	8 (6–13)	6 (4.5–11.5)	0.505
Pulmonary Artery (PA) systolic (mmHg)	54 (40–60)	37 (33–52)	0.153
PA diastolic (mmHg)	28 (21–33)	18 (14–28)	0.092
PA mean (mmHg)	37 (29–42)	22 (19–36)	0.074
Pulmonary capillary wedge pressure (PCWP) (mmHg)	27 (21–28)	22 (18–24)	0.463
Fick Cardiac Output (L/min)	3.67 (3.10–3.90)	5.70 (4.80–7.20)	**0.001**
Fick Cardiac Index (L/min/m^2^)	1.70 (1.50–1.95)	2.80 (2.50–3.00)	**0.001**

Abbreviations: BP, blood pressure; MAP, mean arterial pressure; RA, right atrial; PA, pulmonary artery; PCWP, pulmonary capillary wedge pressure. Data are median (IQR). *p*-values by Wilcoxon signed rank test; N = 15.

**Table 4:** Comparison of KCCQ-12 scores on admission and 2 weeks after Impella 5.5 placement.

Domain	Baseline	2 Weeks Post-Impella	*p*-Value
Symptom Frequency	43.75	70.83	**0.009**
Quality of life	12.5	50	**0.023**
Physical Limitation	50	62.50	0.293
Social Limitation	25	25	0.342
**Overall Summary Score**	**28.13**	**50.52**	**0.005**

Abbreviations: Data are median scores. *p*-values by Wilcoxon signed rank test; N = 15.

## Data Availability

The data presented in this study are available on request from the corresponding author.

## Supplementary Materials

The following supporting information can be downloaded at: https://www.mdpi.com/article/10.3390/medsci13030146/s1, Table S1. KCCQ-12 answers on admission and 2 weeks after Impella 5.5 placement. Data are *n* (%). KCCQ-12 = Kansas City Cardiomyopathy Questionnaire-12, assessing physical limitation, symptom frequency, quality of life, and social limitation. “Limited for other reasons” = unable to perform activity for non-HF reasons. N = 15. Table S2. KCCQ-12 score changes 2 weeks after Impella 5.5 placement. Data are *n* (%). Scores range 0–100; higher = better health status. Change categories: deterioration < –5; no improvement < 5; small > 5–10; moderate > 10–15; large > 15–20; very large > 20. N varies by domain due to missing responses (range 13–15). Table S3. Differences in KCCQ-12 classification on admission and 2 weeks after Impella 5.5 placement. Data are *n* (%). Classification: very poor–poor (0–24), poor–fair (25–49), fair–good (50–74), good–excellent (75–100). Higher = better health status. N varies by domain due to missing responses (range 13–15).

## Abbreviations

ADHF	acute decompensated heart failure
aMCS	axillary mechanical circulatory support
BTT	bridge to transplant
CKD	chronic kidney disease
EF	ejection fraction
HF	heart failure
HFCS	heart failure cardiogenic shock
HfrEF	heart failure with reduced ejection fraction
HRQL	heart related quality of life
KCCQ	Kansas City Cardiomyopathy Questionnaire
LVAD	left ventricular assist device
MCS	mechanical circulatory support
NYHA	New York Heart Association
PROM	patient-reported outcomes measures
QOL	quality of life
REDCap	Research Electronic Data Capture
SRTR	Scientific Registry of Transplant Recipients
