# Supplementary material for: Health-Related Quality-of-Life Measures in Patients with Heart Failure Cardiogenic Shock Following Axillary Mechanical Circulatory Support
Source: Med Sci (Basel). 2025 Aug 19;13(3):146. doi: 10.3390/medsci13030146 (PMC12371966; doi:10.3390/medsci13030146)
Supplement: Supplementary file 1 [file medsci-13-00146-s001.zip › medsci-3767918-supplementary.pdf]

		Before	After
		n (%)	n (%)
Showering/bathing	Extremely limited	6 (40)	5 (33.3)
	Quite a bit Limited	0 (0)	0 (0)
	Moderately Limited	3 (20)	1 (6.7)
	Slightly Limited	4 (26.7)	1 (6.7)
	Not at all Limited	2 (13.3)	4 (26.7)
	Limited for other reasons or did not do the activity	0 (0)	4 (26.7)
Walking 1 block on level ground	Extremely limited	3 (20)	1 (6.7)
	Quite a bit Limited	1 (6.7)	1 (6.7)
	Moderately Limited	3 (20)	4 (26.7)
	Slightly Limited	3 (20)	4 (26.7)
	Not at all Limited	5 (33.3)	3 (20)
	Limited for other reasons or did not do the activity	0 (0)	2 (13.3)
Hurrying or jogging (as if to catch a bus)	Extremely limited	9 (60)	4 (26.7)
	Quite a bit limited	0 (0)	1 (6.7)
	Moderately limited	1 (6.7)	5 (33.3)
	Slightly limited	2 (13.3)	1 (6.7)
	Not at all limited	3 (20)	1 (6.7)
	Limited for other reasons or did not do the activity	0 (0)	3 (20)
Over the past 2 weeks, how many times did you have swelling in your feet, ankles or legs when you woke up in the morning?	Every morning	4 (26.7)	2 (13.3)
	3 or more times per week but not every day	3 (20)	4 (26.7)
	1–2 times per week	3 (20)	1 (6.7)
	Less than once a week	2 (13.3)	3 (20)
	Never over the past 2 weeks	3 (20)	5 (33.3)
Over the past 2 weeks, on average, how many times has fatigue limited your ability to do what you want?	All of the time	4 (26.7)	1 (6.7)
	Several times per day	2 (13.3)	1 (6.7)
	At least once a day	2 (13.3)	2 (13.3)
	3 or more times per week but not every day	4 (26.7)	1 (6.7)
	1–2 times per week	1 (6.7)	3 (20)
	Less than once a week	2 (13.3)	5 (33.3)
	Never over the past 2 weeks	0 (0)	2 (13.3)
Over the past 2 weeks, on average, how many times has shortness of breath limited your ability to do what you wanted?	All of the time	4 (26.7)	1 (6.7)
	Several times per day	3 (20)	1 (6.7)
	At least once a day	1 (6.7)	1 (6.7)
	3 or more times per week but not every day	3 (20)	1 (6.7)
	1–2 times per week	2 (13.3)	3 (20)
	Less than once a week	0 (0)	3 (20)
	Never over the past 2 weeks	2 (13.3)	5 (33.3)
	Every night	4 (26.7)	1 (6.7)

Over the past 2 weeks, on average, how many times have you been forced to sleep sitting up in a chair or with at least 3 pillows to prop you up because of shortness of breath?	3 or more times per week but not every day	2 (13.3)	2 (13.3)
	1–2 times per week	1 (6.7)	1 (6.7)
	Less than once a week	2 (13.3)	2 (13.3)
	Never over the past 2 weeks	6 (40)	9 (60)
Over the past 2 weeks, how much has your heart failure limited your enjoyment of life?	It has extremely limited my enjoyment of life	7 (46.7)	2 (13.3)
	It has limited my enjoyment of life quite a bit	5 (33.3)	3 (20)
	It has moderately limited my enjoyment of life	2 (13.3)	3 (20)
	It has slightly limited my enjoyment of life	0 (0)	3 (20)
	It has not limited my enjoyment of life at all	1 (6.7)	4 (26.7)
If you had to spend the rest of your life with your heart failure the way it is right now, how would you feel about this?	Not at all satisfied	9 (60)	6 (40)
	Mostly dissatisfied	4 (26.7)	4 (26.7)
	Somewhat satisfied	2 (13.3)	5 (33.3)
	Mostly satisfied	0 (0)	0 (0)
	Completely satisfied	0 (0)	0 (0)
Hobbies, recreational activities	Severely limited	8 (53.3)	4 (26.7)
	Limited quite a bit	3 (20)	2 (13.3)
	Moderately limited	2 (13.3)	6 (40)
	Slightly limited	1 (6.7)	0 (0)
	Did not limit at all	0 (0)	1 (6.7)
	Does not apply or did not do for other reasons	1 (6.7)	2 (13.3)
Working or doing household chores	Severely limited	8 (53.3)	5 (33.3)
	Limited quite a bit	2 (13.3)	2 (13.3)
	Moderately limited	3 (20)	3 (20)
	Slightly limited	2 (13.3)	1 (6.7)
	Did not limit at all	0 (0)	0 (0)
	Does not apply or did not do for other reasons	0 (0)	4 (26.7)
Visiting family or friends out of your home	Severely limited	6 (40)	5 (33.3)
	Limited quite a bit	3 (20)	1 (6.7)
	Moderately limited	3 (20)	2 (13.3)
	Slightly limited	0 (0)	0 (0)
	Did not limit at all	1 (6.7)	1 (6.7)
	Does not apply or did not do for other reasons	2 (13.3)	6 (40)

Supplemental Table S1. KCCQ-12 answers on admission and 2 weeks after Impella 5.5 placement. Data are *n* (%). KCCQ-12 = Kansas City Cardiomyopathy Questionnaire-12, assessing physical limitation, symptom frequency, quality of life, and social limitation. "Limited for other reasons" = unable to perform activity for non-HF reasons. N = 15.

		**n (%)**
Summary score change	Deterioration (<-5)	4 (26.7)
	No improvement (<5)	2 (13.3)
	Small improvement (>5)	3 (20)

	Moderate improvement (>10)	0 (0)
	Large improvement (>15)	0 (0)
	Very large improvement (>20)	6 (40)
Physical Limitation change	Deterioration (<–5)	5 (38.5)
	No improvement (<5)	0 (0)
	Small improvement (>5)	2 (15.4)
	Moderate improvement (>10)	1 (7.7)
	Large improvement (>15)	2 (15.4)
	Very large improvement (>20)	3 (23.1)
Symptom Frequency change	Deterioration (<–5)	1 (6.7)
	No improvement (<5)	4 (26.7)
	Small improvement (>5)	1 (6.7)
	Moderate improvement (>10)	0 (0)
	Large improvement (>15)	1 (6.7)
	Very large improvement (>20)	8 (53.3)
Quality of Life change	Deterioration (<–5)	1 (6.7)
	No improvement (<5)	3 (20)
	Small improvement (>5)	0 (0)
	Moderate improvement (>10)	4 (26.7)
	Large improvement (>15)	0 (0)
	Very large improvement (>20)	7 (46.7)
Social Limitation classification change	Deterioration (<–5)	3 (23.1)
	No improvement (<5)	4 (30.8)
	Small improvement (>5)	1 (7.7)
	Moderate improvement (>10)	0 (0)
	Large improvement (>15)	1 (7.7)
	Very large improvement (>20)	4 (30.8)

Supplemental Table S2**.** KCCQ-12 score changes 2 weeks after Impella 5.5 placement. Data are *n* (%). Scores range 0–100; higher = better health status. Change categories: deterioration < –5; no improvement < 5; small > 5–10; moderate > 10–15; large > 15–20; very large > 20. N varies by domain due to missing responses (range 13–15).

		**Before**	**After**
		**n (%)**	**n (%)**
Overall summary score	Very poor to poor	5 (33.3)	2 (13.3)
	Poor to fair	7 (46.7)	5 (33.3)
	Fair to good	3 (20)	6 (40)
	Good to excellent	0 (0)	2 (13.3)
Physical Limitation	Very poor to poor	4 (26.7)	3 (23.1)
	Poor to fair	2 (13.3)	1 (7.7)
	Fair to good	6 (40.0)	5 (38.5)
	Good to excellent	3 (20)	4 (30.8)
Symptom Frequency	Very poor to poor	3 (20)	0 (0)
	Poor to fair	6 (40)	4 (26.7)
	Fair to good	4 (26.7)	4 (26.7)
	Good to excellent	2 (13.3)	7 (46.7)
Quality of Life	Very poor to poor	9 (60)	4 (26.7)
	Poor to fair	4 (26.7)	2 (13.3)
	Fair to good	2 (13.3)	7 (46.7)
	Good to excellent	0 (0)	2 (13.3)
Social Limitation	Very poor to poor	7 (46.7)	6 (46.2)

	Poor to fair	5 (33.3)	1 (7.7)
	Fair to good	3 (20)	5 (38.5)
	Good to excellent	0 (0)	1 (7.7)

Supplemental Table S3. Differences in KCCQ-12 classification on admission and 2 weeks after Impella 5.5 placement. Data are *n* (%). Classification: very poor–poor (0–24), poor–fair (25–49), fair–good (50–74), good–excellent (75–100). Higher = better health status. N varies by domain due to missing responses (range 13–15).
